# Time-to-event analysis to evaluate dormancy status of single-bud cuttings: an example for grapevines

**DOI:** 10.1186/s13007-018-0361-0

**Published:** 2018-10-27

**Authors:** Hector Camargo Alvarez, Melba Salazar-Gutiérrez, Diana Zapata, Markus Keller, Gerrit Hoogenboom

**Affiliations:** 1 AgWeatherNet Program, Irrigated Agriculture Research and Extension Center, Washington State University, Prosser, WA 99350 USA; 2 Department of Horticulture, Washington State University, Prosser, WA 99350 USA; 3 Present Address: Department of Horticulture, Tree Fruit Research and Extension Center, Washington State University, Wenatchee, WA 98801 USA; 4 Present Address: Department of Soil and Crop Sciences, Texas A&M University, College Station, TX 77843 USA; 5 Present Address: Institute for Sustainable Food Systems, University of Florida, Gainesville, FL 32611 USA

**Keywords:** Survival analysis, Phenology, Endodormancy, Paradormancy, Ecodormancy, Budbreak, Chardonnay, Cabernet Sauvignon, *Vitis vinifera* L.

## Abstract

**Background:**

The reduced growth of plants during the winter causes a lack in the perceptibility of the phenological events making challenging the study of dormancy. For deciduous crops, dormancy is generally determined by evaluating budbreak of single-node cuttings that are exposed to conditions suitable for growth. However, the absence of a statistical basis for analyzing and interpreting the budbreak behavior evaluated as the percent budbreak, the average time to budbreak and the time to reach 50% budbreak, has caused inconsistent and contradictory criteria to identify the dormancy status of different deciduous crops.

**Results:**

In this study, a method was developed to analyze the duration between sampling and budbreak of single-node cuttings and to estimate the dormancy status for grapevines (*Vitis vinifera* L.) based on the time-to-event distribution of the observations. This method estimates the probability curve of budbreak for each sample and classifies each curve into paradormancy, endodormancy, and ecodormancy according to the significance when compared to a sample curve estimated from cuttings collected during paradormancy and referred to as “reference.”

**Conclusion:**

The approach described in this study provided a comparison of the budbreak distribution of cuttings collected during distinct phases with a confidence of 95%. It also allowed the estimation of the date of occurrence of the dormancy stages for two grapevine cultivars ‘Cabernet Sauvignon’ and ‘Chardonnay,’ based on the variability within the sampling season rather than on fixed arbitrary criteria. This approach can also be used to analyze budbreak data of single-node cuttings from other common deciduous crops.

**Electronic supplementary material:**

The online version of this article (10.1186/s13007-018-0361-0) contains supplementary material, which is available to authorized users.

## Background

Plant dormancy can be classified into three different phases: paradormancy, endodormancy, and ecodormancy. Paradormancy refers to the period of bud dormancy induced by a structure other than the buds, primarily related to the phenomenon of apical dominance [1, 2]. During the subsequent period; endodormancy, development and growth are controlled by the perception within the bud of an environmental signal. Structures in this state are incapable of growth and development even if the external physiological signals are removed and returned to growth-promoting conditions [2, 3]. Endodormant buds avoid budbreak in response to a transient warming time during late autumn and the subsequent potential damage due to frost. Also, endodormancy is part of the process by which buds adapt to winter conditions and it is a prerequisite for the subsequent acquisition of full cold hardiness [3, 4]. Therefore, endodormancy starts early in autumn prior to leaf senescence [3, 5]. When endodormancy is released, there is a transition to ecodormancy, which is the period preceding budbreak when growth is prevented by one or more environmental factors that are not conducive to growth, but growth is resumed when conditions become favorable again [2].

The synchronism between the growing season, dormancy phase, and the level of cold acclimation can be used as decision support for frost protection management and site and cultivar selection [4, 6]. Nevertheless, it is challenging to research dormancy phenology because of limited activity of the buds and the absence of an on-site method to infer the onset and release of endodormancy [1]. To overcome this limitation, the most commonly used method for estimating the occurrence of endodormancy relies upon the exposure of groups of single-bud cuttings to conditions conducive to growth, known as forcing, with an air temperature around 24 ± 2 °C, a relative humidity between 70 and 90%, and a 15 to 16 h photoperiod [3, 7, 8]. When the elapsed time between sampling and budbreak is long and the percent budbreak is small, buds are endodormant [7]. However, the lack of a statistical basis for analyzing single-node cuttings has resulted in inconsistent criteria and arbitrary thresholds for determining the endodormancy stage [7, 9]. Buds of single-node cuttings of wine grapes under forcing have been classified as endodormant when required between 30 and 50 days to reach 50% budbreak [10, 11], while in a different study endodormancy was identified as the period when 50% of budbreak was reached after 60 days under forcing [12]. Other studies have reported endodormancy as the period when 50% budbreak was reached after 30 days under forcing based on previous reports for apple, sour cherry and peach [13, 14]. However, an average duration to budbreak between 20 and 40 days was found for peach when buds were endodormant [15]. For cherries, 50% budbreak of single-node cuttings was reached after 21 days during endodormancy [16]; and in ‘Sweetheart’ sweet cherry and ‘Gala’ apple, endodormant buds required an average between 20 and 60 days to budbreak [17].

Recent studies have used methods based on time-to-event data or survival analysis to evaluate the depth of dormancy [3, 12, 14, 18]. A parametric approach based on probit models with a log-logistic distribution function was adjusted to budbreak of the single nodes as a function of the time after sampling. The estimated time required to reach 50% budbreak according to this model, a parameter named BR_50_, was used to describe and compare the depth of dormancy of different sampling dates or groups [3, 14]. Non-parametric methods have also been used, such as the Kaplan–Meier estimator of the time-to-event distribution adjusted to the budbreak of single nodes [12, 18]. The Kaplan–Meier estimator has many advantages for evaluating the distribution of budbreak, such as the ease of calculation, the ability to make quantitative estimates without assuming a particular functional distribution, and the capability to compare Kaplan–Meier estimators with statistical significance by a log-rank test. The Kaplan–Meier estimator also considers the presence of right-censored observations, defined as the buds that have not broken at the end of the follow-up period improving the estimation of the budbreak distribution [19–21]. Therefore, the goal of this study was to apply time-to-event analysis to evaluate the budbreak distribution and to estimate the dormancy stage of single-node cuttings of ‘Cabernet Sauvignon’ and ‘Chardonnay.’

## Methods

### Forced single-node cuttings method

Growing shoots and dormant canes of *Vitis vinifera* L. cvs. ‘Cabernet Sauvignon’ and ‘Chardonnay’ were collected starting in August and ending around the first week of December from 2013 to 2016. The samples were obtained from own-rooted grapevines in an experimental vineyard located at the Irrigated Agriculture Research and Extension Center, Washington State University, Prosser, WA (46.3°N; 119.7°W; 355 m above sea level). Meteorological data from the local weather station were provided by the Washington State University Agricultural Weather Network Program (AgWeatherNet, http://weather.wsu.edu). The vines were planted in 2010 in north–south-oriented rows and spaced at 2.7 m between rows and 1.8 m within rows. All vines were cordon-trained, spur-pruned, and drip-irrigated according to regional standard practices.

Samples of shoots were collected weekly every year except in 2014 when shoots were sampled twice per week. Nodes from the third to the fifth basal position were clipped in single-node cuttings. Eighteen cuttings were used per sample during the first 3 years and 30 cuttings were used for the final year. Cuttings were surface-cleaned by soaking them in 70% alcohol for 30 s; they were then washed with distilled water and placed on aluminum trays filled with moist sand substrate. The trays were placed in a growth chamber under forcing conditions at a temperature of 24 ± 2 °C with 15 h of light. Cuttings were observed every 2 days and the number of days between sampling and the appearance of a green leaf tip in the buds, classified in the modified E-L system as stage 4 or budbreak [22], was recorded and defined as the duration to budbreak. Because samples from August were exposed longer to forcing conditions compared to samples from December, duration to budbreak was recorded until the second week of April in 2014, 2015 and 2016 and until the first week of March in 2017 to ensure at least 90 days of exposure to forcing for all the samples. Thus, according to the date of the first sampling each year, the maximum possible duration to budbreak was 215 days for 2013, 224 days for 2014, 238 days for 2015, and 203 days for 2016.

### Data analysis

The mean time required to reach budbreak considering only the buds that broke during the time of evaluation and the percent budbreak were calculated as reported in previous studies for each sample and cultivar [7, 8, 10, 11]. In addition, duration to budbreak of each sample and cultivar, including right-censored observations of the buds that did not break within the follow-up period were adjusted to the survival distribution function by the nonparametric Kaplan–Meier method. This method calculates the probability of the absence of budbreak as a function of time after sampling. The absence of budbreak is defined as the probability that budbreak of one single node does not occur. However, for clarity, the analysis was conducted in terms of the probability of budbreak, which is the complement of the probability of the absence of budbreak.

The first sample of each year of the study, collected in August before the onset of endodormancy, was used as a “reference” of the behavior of budbreak when growth was not restricted within the bud and buds were paradormant. A log-rank test was carried out every year to compare the estimated survival distributions of the reference sample against the other collected samples; a significant difference indicates that the buds were endodormant at the sampling date that was compared to the reference.

### Kaplan–Meier estimator of the survival function

Let *N* represent the number of collected buds and *t*_1_ < *t*_2_ < *t*_3_ < ··· < *t*_*m*_ denote the distinct times at which the buds break or the last day of the follow-up period when there are right-censored observations. The number of recorded dates of budbreak is represented by *m,* and *j* is the index of the observed budbreak times. For each *j *=1,…, *m, m *≤* N* because several buds can break at the same *t*_*j*_, in which case the data contain ties. Let *Y*_*j*_ be the number of buds at risk (not yet broken) just prior to *t*_*j*_ and let *b*_*j*_ be the number of buds broken at *t*_*j*_. Then the Kaplan–Meier estimator Ŝ(*t*) of the survival function is defined as shown in Eq. (1).

1 $${\hat{\text{S}}}\left( t \right) = \mathop \prod \limits_{{j:t_{j} < t}} \left( {1 - \frac{{b_{j} }}{{Y_{j} }}} \right)$$

This means that the function only changes at values of *t* at which $b_{j} \ge 1$ and the censored data are the buds at risk at the end of the follow-up period [19, 23, 24].

### Log-rank test

Let *K* be the number of samples taken during a year and S_*k*_ (*t*) be the survival function of the *k*th sample, *k* = 1,…, *K*. The hypotheses to be tested are:

$$H_{O} {:}\;{\text{S}}_{1} \left( t \right) = {\text{S}}_{2} \left( t \right) = \cdots = {\text{S}}_{K} \left( t \right)\quad{\text{for}}\;{\text{all}}\;t \le \tau ,\;versus$$

$$H_{A} {:}\;{\text{at}}\;{\text{least}}\;{\text{one}}\;{\text{of}}\;{\text{the}}\;{\text{S}}_{\text{k}} (t){\text{'s}}\;{\text{is}}\;{\text{different}}\;{\text{for}}\;{\text{some}}\;t \le \tau$$

where *τ* is the longest time at which all the groups have at least one subject at risk. Let *t*_1_ < *t*_2_ < *t*_3_ < ··· < *t*_D_ be the distinct budbreak times from the pooled sample [24, 25]. Let $b_{i} = \sum\nolimits_{k = 1}^{K} {b_{ik} }$ be the number of budbreak in the pooled sample at time *t*_*i*_, *i *= 1,…, D, and let $Y_{i} = \sum\nolimits_{k = 1}^{K} {Y_{ik} }$ be the number of buds at risk in the combined sample at time *t*_*i*_ [23, 24]. The standard log-rank test is based on the statistic *G*, which has the form of a K-vector $G = \left( {G_{1} , . . . , G_{K} } \right)$ with:

2 $$G_{k} = \mathop \sum \limits_{i = 1}^{D} \left( {b_{ik} - Y_{ik} \frac{{b_{i} }}{{Y_{i} }}} \right)$$

The variance of $G_{k}$ is $V_{kk}$ as defined for Eq. (3) and the covariance between $G_{k}$ and $G_{h}$ statistics calculated for the *k*th and *h*th samples is $V_{kh}$ shown in Eq. (4).

3 $$V_{kk} = \mathop \sum \limits_{i = 1}^{D} \frac{{Y_{ik} }}{{Y_{i} }}\left( {1 - \frac{{Y_{ik} }}{{Y_{i} }}} \right)\left( {\frac{{Y_{i} - b_{i} }}{{Y_{i} - 1}}} \right)b_{i} , \quad 1 \le k \le K$$

4 $$V_{kh} = \mathop \sum \limits_{i = 1}^{D} \frac{{Y_{ik} }}{{Y_{i} }}\frac{{Y_{ih} }}{{Y_{i} }}\left( {\frac{{Y_{i} - b_{i} }}{{Y_{i} - 1}}} \right)b_{i} , \quad 1 \le k \ne h \le K$$

Let *V* be the estimated variance–covariance matrix formed by the $V_{kk}$ and $V_{kh}$ values from Eqs. (3) and (4). Thus, a *K*-sample test for *H*_*O*_ versus *H*_*A*_ has a Chi squared distribution ($\upchi^{2}$) and can be calculated as shown in Eq. (5).

$$\upchi^{2} = \left( {G_{1} , \ldots ,G_{K} } \right)V^{ - } \left( {G_{1} , \ldots ,G_{K} } \right)' \text{with K-1 degrees of freedom, and a}\, p \,\text{value}$$

5 $$p = \Pr (\upchi^{2} >\upchi^{2} )$$

When the comparison is performed only between two samples, as in this study when each individual survival distribution was compared against the reference, a two-sided test statistic that compares the *k*th against the *h*th samples is used as shown in Eq. (6) and the hypotheses to be tested are:

$$H_{O} {:}S_{k} \left( t \right) = S_{h} \left( t \right)\quad {\text{for all}}\;t \le \tau ,\;versus$$

$$H_{A} {:}S_{k} \left( t \right) \ne S_{h} \left( t \right)\quad{\text{for}}\;{\text{some}}\;t \le \tau$$

6 $$z_{kh}^{2} = \frac{{\left( {G_{k} - G_{h} } \right)^{2} }}{{V_{kk} + V_{hh} - 2V_{kh } }}$$

$$p = \Pr (\varvec{\chi}^{2} > z_{kh}^{2} )\quad 1\;{\text{degree}}\;{\text{of}}\;{\text{freedom}}$$

The *K*-1 samples collected for 1 year were compared against the reference for that year. Then, *K*-1 two-sample hypotheses were tested simultaneously in a multiple comparison test. When multiple comparisons are performed, the family-wise error rate (FWE), which is the probability of incorrectly rejecting one or more of the *K*-1 hypotheses simultaneously, is the controlled α rather than the comparison wise error (CWE), which is the probability of incorrectly rejecting a single comparison or hypothesis. The CWE should be less than or equal to the FWE in order to obtain the exact coverage probability 1-α [24, 26, 27]. Thus, simultaneous p-values for all comparisons were adjusted and scaled according to the variance–covariance matrix using the Dunnett-Hsu adjustment, which increases the ${\varvec{\upchi}}^{2}$ critical value of rejection and consequently decreases the CWE for every single comparison for an FWE α = 0.05 [23]. The statistical analysis was conducted using the SAS software version 9.4 with PROC LIFETEST (SAS Institute, Cary, NC) [23].

**Fig. 1:**
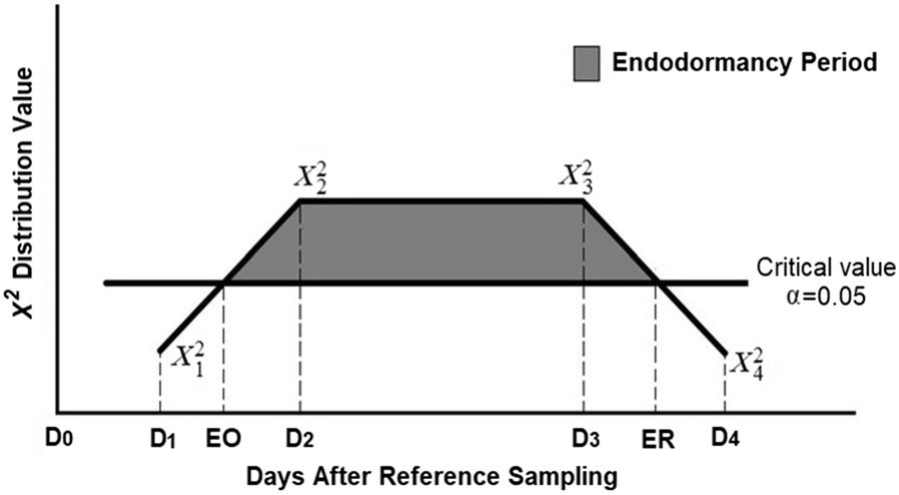
Representation of the linear interpolation for estimation of the dates of endodormancy onset (EO) and endodormancy release (ER). The vertical axis represents the X^2^-value of the comparison between the sample taken at date D*i* against the reference sample taken at D0, with a family-wise error (FWE) α = 0.05

The ${\varvec{\upchi}}^{2}$ values of the comparisons between each sample and the reference were sorted chronologically every year for each cultivar and a straight line was projected between the ${\varvec{\upchi}}^{2}$ values of the first sample with a significant difference and the preceding sample. The onset of endodormancy was estimated as the date when the line crossed the adjusted critical ${\varvec{\upchi}}^{2}$ value for hypothesis rejection with 95% confidence as defined in Eq. (7). Similarly, the release of endodormancy was estimated as the date of the crossing of the adjusted critical ${\varvec{\upchi}}^{2}$ value with 95% confidence and the straight line obtained between the ${\varvec{\upchi}}^{2}$ values of the last sample with a significant difference and the subsequent sample as defined in Eq. (8). This process was conducted with the Solver tool of Microsoft Office Excel Version 2013.

7 $$EO = \frac{{\left( {D2 - D1} \right)\left( {Cv - {\varvec{\upchi}}_{1}^{2} } \right)}}{{\left( {{\varvec{\upchi}}_{2}^{2} - {\varvec{\upchi}}_{1}^{2} } \right)}} + D1$$

8 $$ER = D4 - \frac{{\left( {D4 - D3} \right)\left( {Cv - {\varvec{\upchi}}_{4}^{2} } \right)}}{{\left( {{\varvec{\upchi}}_{3}^{2} - {\varvec{\upchi}}_{4}^{2} } \right)}}$$

where *EO* is the onset of endodormancy, *ER* is the release of endodormancy, *D1* is the date of the sample preceding *D*2, which is the date of the first sample with significant difference relative to the reference, *D*3 is the last date with significant difference against the reference, and *D*4 is the date of the subsequent sample. ${\varvec{\upchi}}_{\varvec{i}}^{2}$ is the Chi squared value of the comparison between the sample from each *Di* and the reference, and *Cv* is the adjusted critical value corresponding to an FWE α = 0.05 (Fig. 1). The computation of adjusted critical values requires arduous calculations [26]. Therefore, in our study computations were made mathematically using either Microsoft Office Excel version 2013 or SAS software version 9.4 with PROC LIFETEST (SAS Institute, Cary, NC) as described in the Additional files 1 and 2.

## Results

### Average duration to budbreak and percent budbreak

**Fig. 2:**
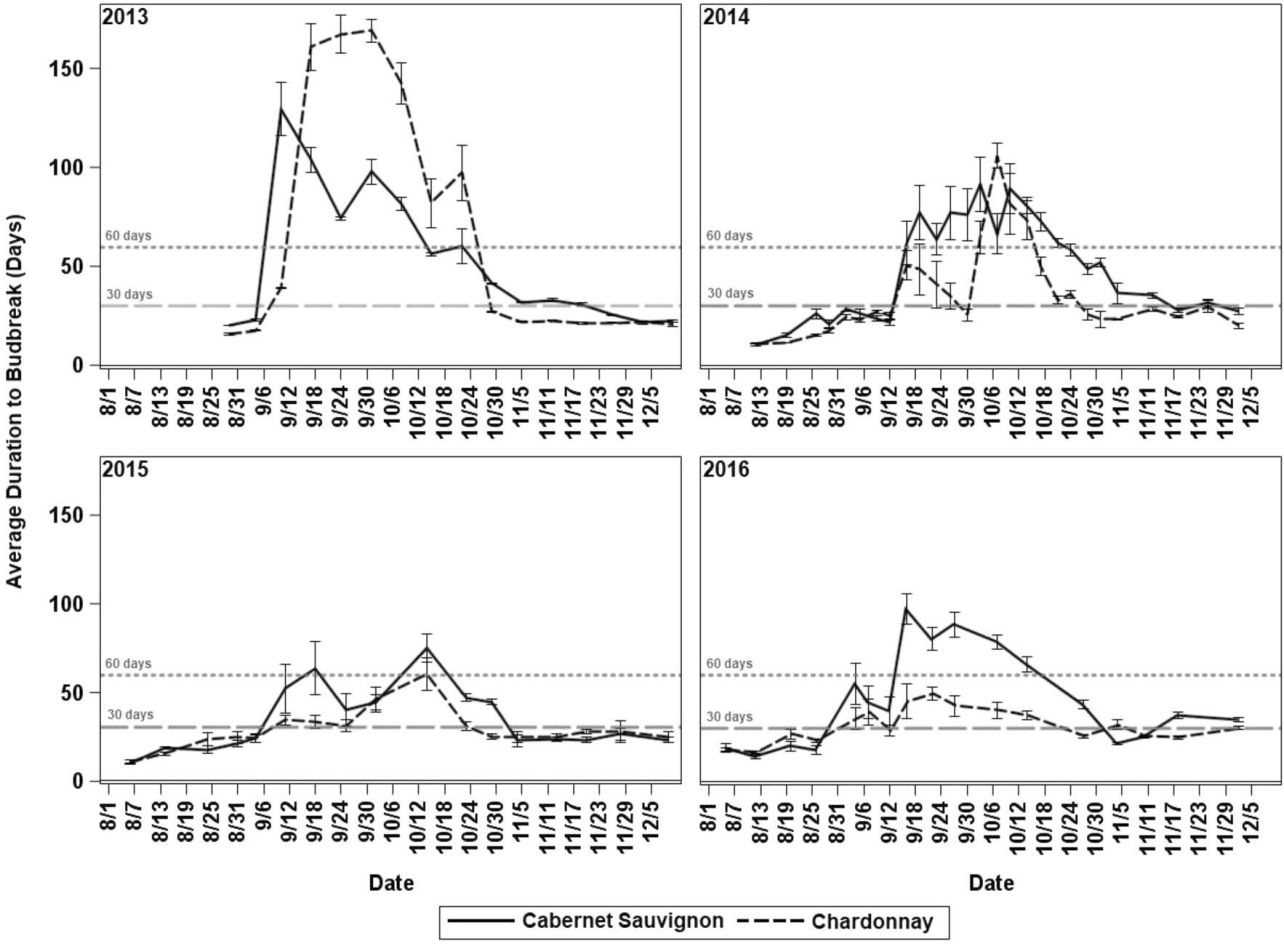
Average duration to budbreak and standard error of single node cuttings from each sample calculated only with buds that broke during the evaluation period for ‘Cabernet Sauvignon’ and ‘Chardonnay’ grapevines in Prosser, Washington, for 2013, 2014, 2015, and 2016. 60 and 30 days lines represent thresholds previously used in literature

**Fig. 3:**
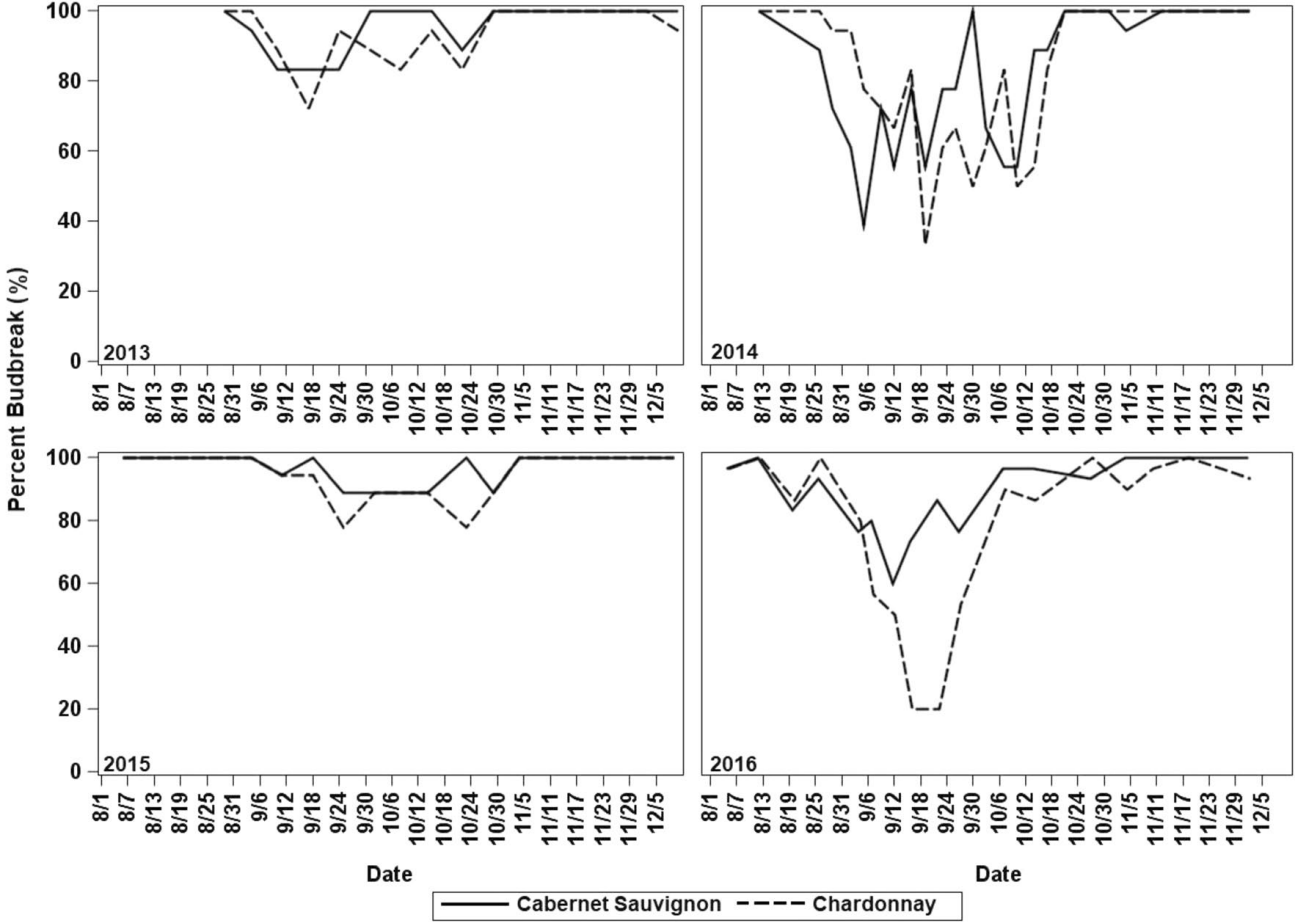
Percent budbreak of each sample during the evaluation period, calculated from 18 single node cuttings (30 in 2016) for ‘Cabernet Sauvignon’ and ‘Chardonnay’ in Prosser, Washington, for 2013, 2014, 2015, and 2016

Throughout the 4 years of the evaluation, both grapevine cultivars showed a period of increase of the average duration to budbreak and reduction of the percent budbreak of the cuttings caused by an internal arrest of bud growth during endodormancy. However, the onset and the end of these events did not coincide during each year. In 2013, for ‘Cabernet Sauvignon’ the average duration to budbreak increased sharply from 23 to 130 days at 4 September and decreased at the end of October, suggesting the end of endodormancy, while the percent budbreak decreased from 100% to 83% only during September. In 2014 and 2016, the increase of the average duration to budbreak occurred between the first and second weeks of September and lasted until the first week of November, while the percent budbreak dropped from mid-August to mid-October. The increase of the average duration to budbreak and reduction of the percent budbreak occurred simultaneously only in 2015 starting the first week of September and ending during the last week of October (Figs. 2, 3).

For ‘Chardonnay,’ the duration to budbreak in 2013 increased sharply from 18 to 170 days from 4 September until mid-October, when it declined rapidly to 22 days. At the same time, budbreak dropped from 100% to values around 75%. Likewise, in 2016 the duration to budbreak reached values around 100 days, and simultaneously the percent budbreak dropped to 20% from the first week of September to mid-October. During 2014 and 2015, the average duration to budbreak increased more slowly reaching approximately 100 days in 2014 and 60 days in 2015 starting the second week of September until the last week of October. However, in 2014 the percent budbreak decreased from the first week of September until mid-October reaching a minimum of 30%, while in 2015, the decline in budbreak occurred from mid-September until the first week of November, reaching a minimum of 70% (Figs. 2, 3).

### Kaplan–Meier estimator and log-rank test

Although there were marked differences between years in the duration to budbreak, the estimated Kaplan–Meier survival functions (curves of probability of budbreak) and the log-rank test showed similar patterns every year with three different periods. The reference curve, estimated with paradormant buds, was characterized by a sharp increase in the probability of budbreak during the first 50 days under forcing, with limited to no presence of right-censored observations. The survival curves for the subsequent samples showed a similar response and were not significantly different from the reference curve, suggesting that the buds were still paradormant. The second period was characterized by curves with a slow increase of the probability of budbreak, taking longer than 100 days under forcing for some buds to break. Additionally, during this period there was a higher number of right-censored observations and the samples in this period had probability curves significantly different from the reference curve, suggesting that the buds were endodormant. Finally, during the third period, related to ecodormancy the curves were not significantly different relative to the reference curve, and endodormancy had already occurred.

**Fig. 4:**
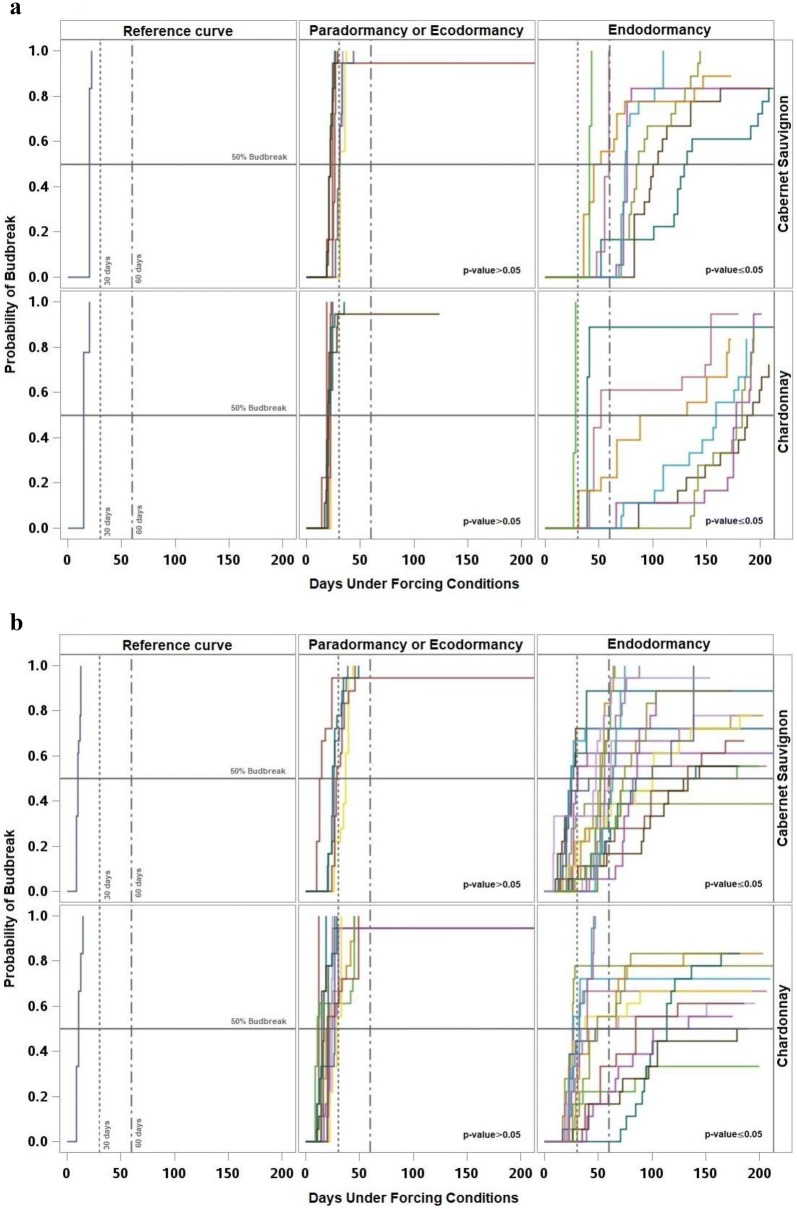
Kaplan-Meier survival curves of the reference sample (left), sampling dates with no significant difference against the reference classified as paradormant or ecodormant (middle), and sampling dates with significant difference against the reference classified as endodormant (right), for ‘Chardonnay’ (bottom) and ‘Cabernet Sauvignon’ (top) for 2013 (**a**), 2014 (**b**)

**Fig. 5:**
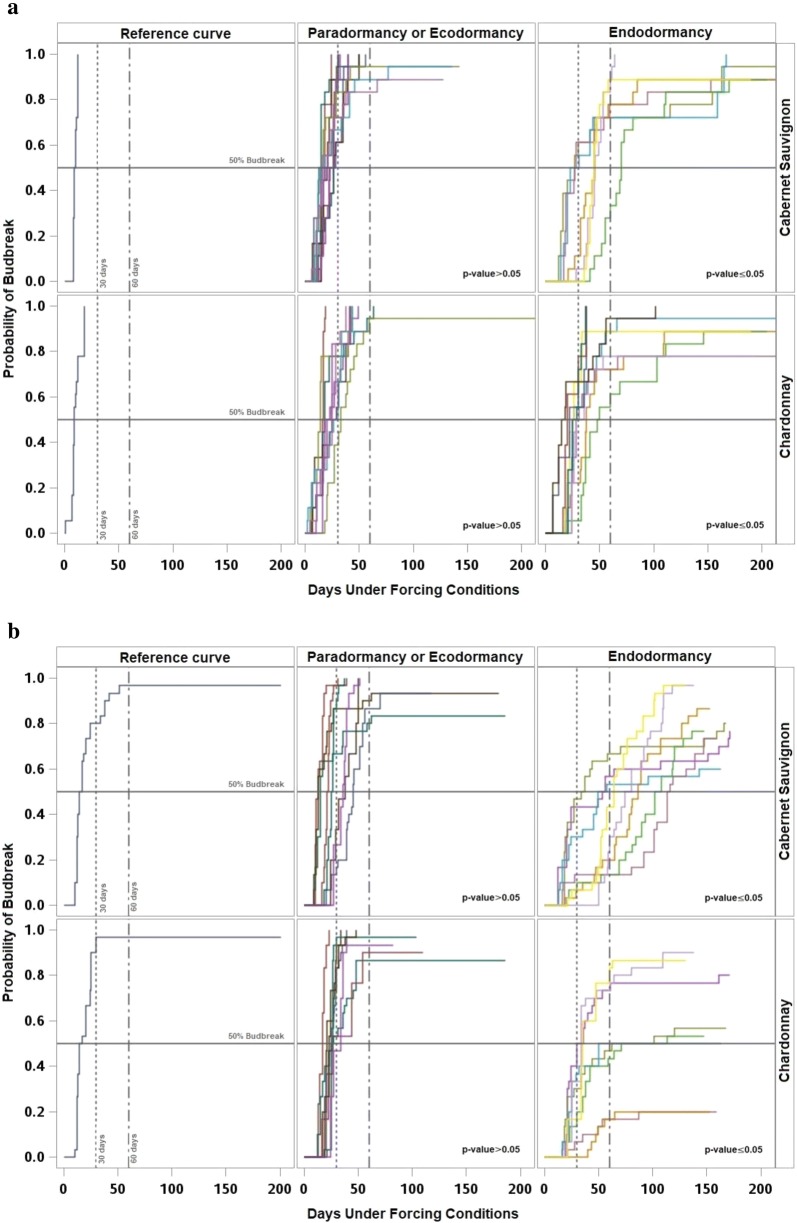
Kaplan-Meier survival curves of the reference sample (left), sampling dates with no significant difference against the reference classified as paradormant or ecodormant (middle), and sampling dates with significant difference against the reference classified as endodormant (right), for ‘Chardonnay’ (bottom) and ‘Cabernet Sauvignon’ (top) for 2015 (**a**), and 2016 (**b**)

Each year for both cultivars, 50% percent of budbreak of the reference sample occurred before 30 days under forcing. Similarly, most of the samples that were not significantly different from the reference required fewer than 30 days under forcing conditions to reach 50% budbreak, with the exception of one sample in 2015 and one sample in 2016 for ‘Chardonnay,’ and three samples in 2013, one sample in 2014 and three samples in 2016 for ‘Cabernet Sauvignon.’ Consequently, most of the samples for both cultivars that showed significant differences against the reference reached 50% budbreak after 30 days under forcing. Except for three samples of each cultivar during 2014, and two samples of ‘Cabernet Sauvignon’ and six samples of ‘Chardonnay’ during 2015 which had numerous right-censored observations (Figs. 4, 5).

In 2013, eight consecutive samples for both cultivars were identified as endodormant starting on 9 September and ending on 29 October. Although all the curves with significant differences reached 50% budbreak, in five samples of ‘Chardonnay’ it required more than 100 days (Fig. 4a). In 2014, 21 samples of ‘Cabernet Sauvignon’ between 26 August and 4 November and 16 samples of ‘Chardonnay’ between 2 September and 24 October were identified as endodormant. The samples collected in 2014 showed numerous curves with right-censored observations; 12 curves in ‘Cabernet Sauvignon’ and 11 curves in ‘Chardonnay’ had a budbreak lower than 80% (Fig. 4b). In 2015, ‘Cabernet Sauvignon’ had eight successive samples between 4 September and 29 October, and ‘Chardonnay’ had seven samples between 11 September and 29 October identified as endodormant. The samples collected in 2015 had the lowest amount of right-censored observations; all the curves had percent budbreak equal to or greater than 80% (Fig. 5a). In 2016, eight consecutive sampling dates between 4 September and 14 October for both cultivars were identified as endodormant and none of the curves reached 100% budbreak (Fig. 5b).

### Onset of endodormancy and release date estimation

**Fig. 6:**
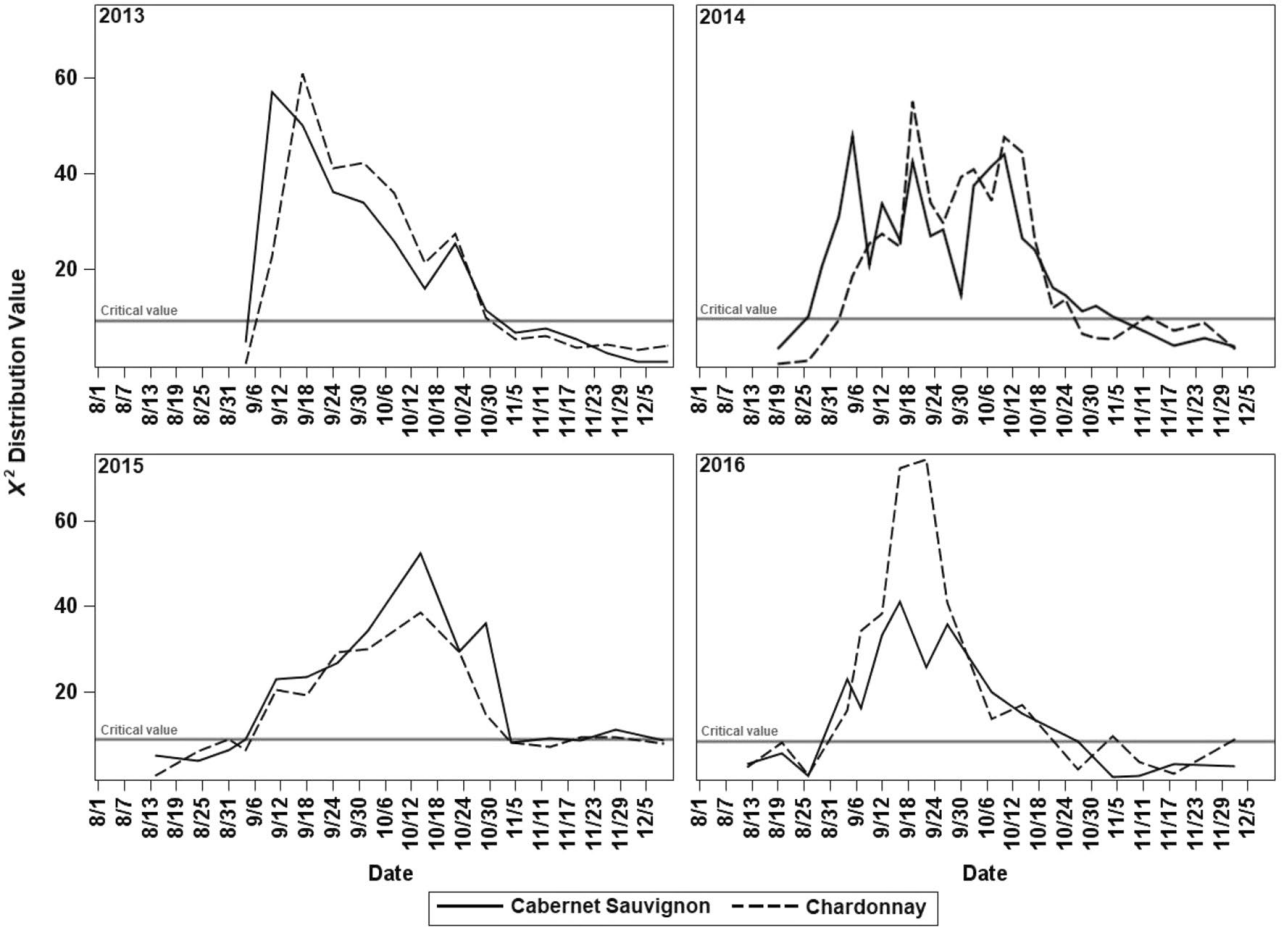
Chi squared values of the log-rank comparison between the survival function of each sample against the survival function of the reference sample (non-dormant first sample of every year) for 2013, 2014, 2015, and 2016. The solid line represents the $\chi ^{2}$ critical value with an FWE α = 0.05

The ${\varvec{\upchi}}^{2} \varvec{ }$ values of the comparison between the survival curves of the reference and the remainder of the samples were higher than the critical value of rejection, with an FWE of α = 0.05 during endodormancy; the higher the Chi squared value, the deeper the dormancy. In 2013, the most profound dormancy occurred between 7 and 10 September for both cultivars; in 2014 it occurred on 5 September for ‘Cabernet Sauvignon’ and 19 September for ‘Chardonnay”; for 2015, the most profound dormancy occurred on 14 October for both cultivars; and for 2016 it occurred on 16 September for ‘Cabernet Sauvignon’ and between 16 and 22 September for ‘Chardonnay’ (Fig. 6). The estimated onset of endodormancy occurred slightly later each year for ‘Chardonnay’ between 31 August and 6 September, compared to ‘Cabernet Sauvignon,’ which became endodormant between 26 August and 4 September. In contrast, the estimated release of endodormancy occurred earlier for ‘Chardonnay,’ between 21 October and 3 November, while for ‘Cabernet Sauvignon’ it occurred between 27 October and 5 November. As a result, ‘Chardonnay’ had a shorter endodormancy period than ‘Cabernet Sauvignon’ for each year of the study period (Table 1).

**Table 1:** Estimated dates for the onset and release of endodormancy for ‘Cabernet Sauvignon’ and ‘Chardonnay’ in Prosser, WA

Cultivar	Year			
	2013	2014	2015	2016
Cabernet Sauvignon				
Onset	4-Sep	26-Aug	4-Sep	29-Aug
Release	2-Nov	5-Nov	4-Nov	27-Oct
Chardonnay				
Onset	6-Sep	2-Sep	5-Sep	31-Aug
Release	31-Oct	26-Oct	3-Nov	21-Oct

## Discussion

During the 4 years of the study, a period characterized by the simultaneous increase of the average duration to budbreak and reduction of percent budbreak of nodes exposed to forcing was quickly identified as endodormancy [10, 11]. In contrast, the typical response of the buds during paradormancy and ecodormancy was characterized by a low average duration to budbreak and simultaneous budbreak close or equal to 100% [12, 13]. However, when the response of the duration to budbreak and percent budbreak was not as clear, generally around the transitions from paradormancy to endodormancy and subsequently to ecodormancy, the determination of the dormancy phase was challenging. An additional limitation was caused by the temporal differences between both variables as found during 2013, 2014, and 2016 in ‘Cabernet Sauvignon’ and during 2014 and 2015 in ‘Chardonnay,’ when the percent budbreak tended to decrease 1 or 2 weeks earlier than the increase of the average duration to budbreak. These temporal differences can be caused by the early induction of endodormancy in a small percentage of buds following the regular cumulative frequency of any phenological stage [28], the genetic variation within the grapevine, variability in bud microclimate, and variability in the distance from the shoot base [29, 30]. These factors affect the percent budbreak earlier than the average duration of budbreak. In addition, the range of duration to budbreak and percent budbreak varied every year suggesting that duration to budbreak and percent budbreak cannot be analyzed separately and both variables are complementary to identify endodormancy. For example, during 2014 and 2016 ‘Chardonnay’ had samples with a duration to budbreak that was less than 50 days but a very low percent budbreak, while in 2013 the opposite occurred and the percent budbreak was higher than 80% and the duration to budbreak was greater than 100 days.

The annual variation in the range of duration to budbreak and the percent budbreak also suggests that the estimations of the dormancy phase based on fixed, arbitrary time thresholds to evaluate 50% of budbreak, such as 30 days under forcing [13] and 60 days under forcing [12], in addition to being inconsistent are very imprecise, especially if many buds are right-censored. When the average duration to budbreak or the time required by the sample to reach 50% budbreak are compared to these thresholds, the right-censored observations are counted as null, ignoring useful information [8, 9, 17]. On the other hand, the limits at which the duration to budbreak and percent budbreak start to be ‘high’ or ‘low’ are not fixed; they are affected each year by the response of the cultivar to experimental conditions and weather variability [8]. For instance, differences of only 1 °C in the forcing temperature can impact the duration to budbreak of the cuttings [31]. Additionally, temperatures before bud sampling can affect the bud response under forcing conditions. Thus, warm air temperatures during shoot development in *V. vinifera* stimulate axillary-bud outgrowth, modifying the duration to budbreak of the cuttings [32]. Also, elevated temperatures during dormancy induction can cause slow and shallow dormancy as found in *Populus* x spp [33].

When the results of this study were analyzed based on time-to-event distributions, the analysis dealt with the synchronicity problem between duration to budbreak and percent budbreak, integrating both variables to estimate the curves of probability of budbreak (Figs. 4, 5). Also, the Kaplan–Meier survival functions and the log-rank test for comparison of survival curves with a 95% of confidence allowed the occurrence estimation of the transitions between paradormancy, endodormancy and ecodormancy. These estimates were based on the variability of the samples under the environmental conditions at which plants grew and experimental conditions at which budbreak was induced, increasing the precision of the estimation related to the fixed threshold methods. The results of the log-rank test were partially consistent with the results found when paradormancy and ecodormancy were determined as the period when 50% budbreak occurred before 30 days, mainly for ‘Chardonnay.’ However, the results were less comparable to determine endodormancy when there was a large number of right-censored observations, such as in 2014 and 2016. This discrepancy caused samples classified as endodormant by the log-rank test to be classified as paradormant or ecodormant by the fixed threshold method because they showed 50% budbreak before 30 days under forcing, but the sample only reached between 60 and 70% budbreak. Also, in some samples a 50% budbreak was never reached, making the response not interpretable with the fixed threshold method. The results of the log-rank test did not show any relation to the 60-day threshold.

The estimated onset of endodormancy occurred consistently between 1 and 6 days earlier each year for ‘Cabernet Sauvignon’ compared to ‘Chardonnay’ (Table 1). This difference is caused by the ecotypic variation in sensing the triggering signals for the induction of endodormancy, such as photoperiod, temperature, light intensity, and water status [4, 5]. Differences in onset of endodormancy presented in this study diverge with previous results where the onset of endodormancy for ‘Cabernet Sauvignon’ and ‘Chardonnay’ coincided [12]. However, those results were based on monthly and bi-weekly samplings, and thus the difference between cultivars could have been overlooked.

**Fig. 7:**
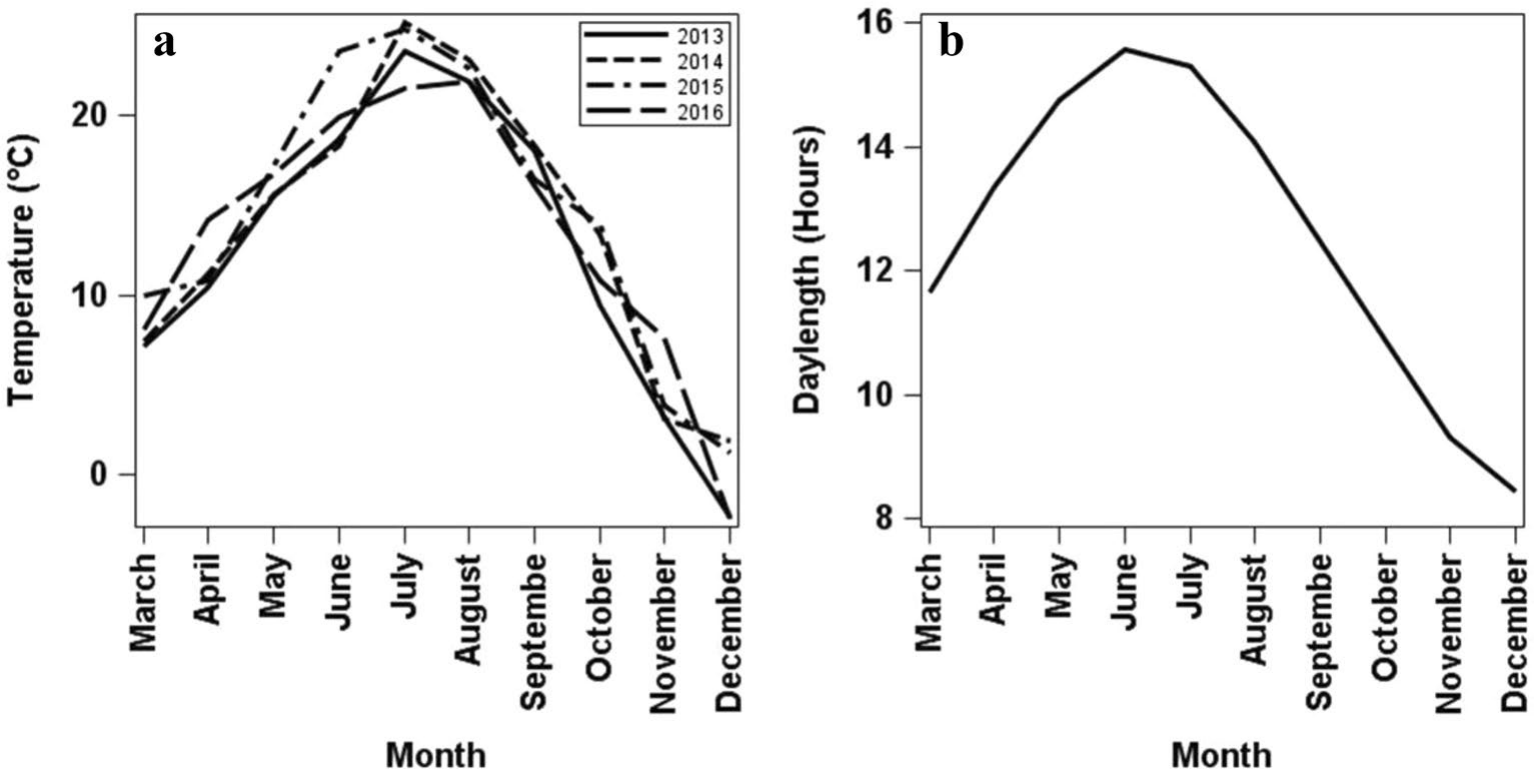
Environmental conditions in Prosser, Washington, during the growing and dormancy periods for the 4 years of the study. **a** Monthly average temperature. **b** Daylength of the 21st day of each month

The dates estimated by the interpolation method occurred when the photoperiod at Prosser, Washington, where this study was conducted ranged between 13.1 and 13.5 h for ‘Cabernet Sauvignon’ and between 13.0 and 13.3 h for ‘Chardonnay’ (Fig. 7). These day lengths are similar to those that promote the initiation of budbreak in *V. riparia* with 13 h, and *V. labruscana* with 12 h [34]. Species with photoperiodism initiate endodormancy approximately the same day each year when the critical daylength is exceeded [35]. However, differences in the estimated dates of onset of endodormancy among years found in this study suggest that temperature may modulate this onset as reported for *V. vinifera* cv. ‘White Riesling’ where the interaction between photoperiod and low temperatures enhanced the onset of endodormancy [36, 37]. Additionally, the estimated onset of endodormancy occurred earlier in 2014 and 2016 for both cultivars, with 2014 having the warmest and 2016 having the lowest temperatures during August before the onset of endodormancy (Fig. 7). This response could be related to the recently proposed existence of two different pathways of temperature-mediated dormancy induction in woody plants, i.e., a low temperature-induced stress pathway in northern latitude ecotypes, and a warm night temperature-short photoperiod induced pathway that affects all ecotypes [33]. Although this model is still unclear, these pathways may explain the plasticity of dormancy induction development and the adaptations of plants to ensure dormancy development under variable autumn temperatures, thereby maximizing their growing season [9, 33].

The estimated dates of release of endodormancy occurred between 21 October and 5 November for all years (Table 1); consistent with previous reports for buds of *V. vinifera* which were already ecodormant during November and December [6, 12]. The variation in the release of endodormancy between years is mainly related to the chilling requirement, which is the amount of temperature below a threshold required to satisfy endodormancy and to advance to ecodormancy [6, 18]. While the relationship between chilling temperatures and estimated dates of endodormancy release is beyond the scope of this study, it is nonetheless, challenging to determine as the chilling requirement of grapevines is not well described. Grapevines require a low exposure to chilling compared to many other deciduous fruit crops like apple or peaches, and chilling has even been considered a facultative rather than an absolute requirement [11, 38]. Trials under controlled conditions have reported different thresholds of chilling accumulation. For instance, 11.5 °C for ‘Cabernet Sauvignon’ and ‘Chardonnay’ [6], 6 °C for ‘Cabernet Sauvignon’ [39] and 3 °C for ‘Cabernet Sauvignon’ and − 3 °C for ‘Chardonnay’ were reported as the temperature with the highest efficiency of chilling [12]. Furthermore, chilling thresholds estimated from field data have been inconsistent in physiological terms, showing chilling efficiency at temperatures as high as 30 °C for ‘Sangiovese’ and 20 °C for ‘Chardonnay’ [38, 40]. Differences in the release of endodormancy between cultivars were also found; the estimated dates of ‘Chardonnay’ occurred consistently between 1 and 10 days earlier compared to ‘Cabernet Sauvignon.’ Previous reports under controlled conditions also found an earlier transition from endodormancy to ecodormancy in ‘Chardonnay’ and a higher requirement of chilling units in ‘Cabernet Sauvignon’ compared to ‘Chardonnay’ using the same threshold of 11.6 °C for both cultivars, causing a more extended endodormancy period in the former cultivar [6, 12].

## Conclusion

The use of the Kaplan–Meier estimator of the survival function and the log-rank test with a FWE α = 0.05 allowed the analysis of the budbreak distribution of forced single-node cuttings and the estimation of the occurrence of the dormancy phases and the dates of transitions for two grapevine cultivars ‘Cabernet Sauvignon’ and ‘Chardonnay,’ following the statistical nature of the time-to-event data and based on the variability within the sampling season rather than on arbitrarily fixed thresholds. The estimated dates were consistent with previous reports and the method could be used to analyze the single-node cuttings results commonly obtained in other deciduous crops.

## Additional files

**Additional file 1.** Mathematical estimation of critical values of χ^2^ corresponding to a FWE α = 005 using Microsoft Office Excel version 2013.

**Additional file 2.** Computation of critical values of χ^2^ corresponding to a FWE α = 0.05 using SAS software version 9.4.

## Abbreviations

FWE: family-wise error
CWE: comparison-wise error
